# The preliminary evidence on the association of the gut microbiota with stroke risk stratification in South Chinese population

**DOI:** 10.3389/fcimb.2023.1227450

**Published:** 2023-12-21

**Authors:** Haiyan Huang, Zhuoran Kuang, Ruibi Mo, Miaomiao Meng, Yefeng Cai, Xiaojia Ni

**Affiliations:** ^1^ The Department of Neurology, Guangdong Provincial Hospital of Chinese Medicine, Guangzhou, China; ^2^ The Second Clinical School of Guangzhou University of Chinese Medicine, Guangzhou, China; ^3^ Guangdong Provincial Academy of Chinese Medical Sciences, Guangzhou, China; ^4^ State Key Laboratory of Dampness Syndrome of Chinese Medicine, The Second Affiliated Hospital of Guangzhou University of Chinese Medicine, Guangzhou, China

**Keywords:** gut microbiota, the risk of stroke, 16S rRNA sequencing, lipid metabolism, inflammation, atherosclerosis

## Abstract

**Aims:**

This study aimed to investigate the association between the gut microbiota and the risk of stroke.

**Methods:**

Faecal samples from 60 participants in South China, including 45 individuals with risk factors for stroke and 15 healthy controls, were collected and subjected to 16S rRNA sequencing. A bioinformatics analysis was performed to characterise the gut microbial diversity and taxonomic compositions at different risk levels (low, moderate, and high) of stroke. Functional prediction and correlation analyses between the microbiota and laboratory markers were performed to explore the potential mechanisms.

**Results:**

A significant difference in beta diversity was observed between the participants from the stroke risk and healthy control groups. Linear discriminant effect size analysis revealed a large number of vascular beneficial bacteria enriched in the participants from the healthy control and low-risk groups, but a few vascular harmful bacteria were more abundant in the participants from the high-risk group than in those from the other groups. In addition, *Anaerostipes*, *Clostridium_XlVb*, and *Flavonifractor*, all of which belonged to the *Firmicutes* phylum, were enriched in the participants from the low-risk group, and their relative abundances gradually decreased as the stroke risk increased. Spearman’s analysis revealed that these outstanding microbiota correlated with the levels of triglycerides, high-density lipoprotein cholesterol, low-density lipoprotein cholesterol, white blood cells, neutrophils, and carotid intima-media thickness.

**Conclusion:**

The preliminary evidence suggests that gut microbiota is associated with stroke risk. It potentially ameliorates atherosclerosis by targeting lipid metabolism and inflammation. This provides novel insights into the early screening of stroke risk and primary prevention.

## 1 Introduction

Stroke remains the second leading cause of death and the third leading cause of death and disability worldwide (Feigin et al., 2022). From 1990 to 2019, the absolute number of stroke cases increased substantially (i.e. 70.0% increase in incident stroke rates), and over 12.2 million new stroke cases were reported each year, thereby necessitating urgent measures to reduce stroke incidence (GBD 2019 Stroke Collaborators, 2021). In China, stroke is the leading cause of disability-adjusted life year, and 3.94 million new stroke cases were recorded in 2019 (Wang et al., 2022). Traditionally, identification and modification of risk factors are essential for reducing stroke incidence (Pandian et al., 2018). However, considerable residual risks of stroke remain as conventional risk factors, which account for 90% risk of stroke worldwide and in China (Qi et al., 2020). Thus, treating the arteries instead of reducing the risk factors has been suggested (Spence and Hackam, 2010).

Atherosclerosis, characterised by lipid accumulation and inflammation of the large arteries, is a major cause of stroke (Björkegren and Lusis, 2022). Dysbiosis, which refers to microbial imbalance or dysfunction in the gut, has attracted considerable attention because of its role in atherosclerosis development (Tang et al., 2019). The gut microbiota and its metabolites may affect the risk factors of stroke, such as hypertension, obesity, and diabetes, or signal inflammation and immune responses during atherosclerosis and thrombosis (Wang et al., 2022). To be specific, a growing body of evidence suggests that *Trimethylamine-N-oxide (TMAO)*, a small molecular compound derived from the metabolism of intestinal microorganisms, is associated with the elevated risk of stroke, as well as the severity and prognosis of stroke (Liu et al., 2023). Clinical evidence from dose-response meta-analyses also show the circulating TMAO is positively associated with an increased probability of risk factors in stroke, such as hypertension (Abbasalizad Farhangi and Vajdi, 2021), and inflammatory parameters involved in the pathological process of atherosclerosis, such as C-reactive protein (Farhangi and Vajdi, 2020). Besides, the mediation of the therapeutic effects and adverse events by the gut microbiome has been identified in the prevention and treatment of stroke such as statins and aspirin (Chakaroun et al., 2023).

The primary prevention strategy tailored to stroke risk stratification instead of individual risk factors has been developed, and included to clinical practice guidelines (Meschia et al., 2014; Arnett et al., 2019; Owolabi et al., 2022). However, few studies have investigated the association between the gut microbiome and stroke risk stratification. Thus, this study aimed to determine the association between the gut microbiota and stroke risk stratification.

## 2 Materials and methods

### 2.1 Ethics approval

This study was conducted in accordance with the Declaration of Helsinki (2013) and approved by The Ethics Committee of Guangdong Provincial Hospital of Chinese Medicine (institutional approval number No. BE2021-195). Each participant signed a written informed consent form.

### 2.2 Study Population

All the participants were of Chinese descent long residing in China. Hence, the stroke risk factors identified by the Ministry of Health China Stroke Prevention Project Committee were adopted in this study (Chao et al., 2021). The participants of this study were recruited from a cross-sectional survey of a population with risk factors of stroke. The inclusion criteria were as follows: 1) age ≥40 years, 2) living in Guangdong Province for more than half a year, and 3) having one or more than one risk factors of stroke, including hypertension, diabetes mellitus, atrial fibrillation, smoking, dyslipidaemia, physical inactivity, overweight/obesity, and a family history of stroke, or a medical history of transient ischaemic attack (TIA) or stroke. Individuals aged ≥40 years and without any risk factor of stroke or a history of TIA/stroke were recruited from the same community-based population living in Guangdong Province for more than 6 months as healthy controls. The criteria for the individual risk factors are specified in **Supplementary Table 1**.

Participants were excluded if they met one of the following criteria: 1) diagnosis of a severe mental disorder or cognitive impairment affecting the face-to-face survey, 2) diagnosis of a major gastrointestinal disorder, and 3) ingestion of oral antibiotics, probiotics, coffee, wine or other fermented foods (e.g. traditional Asian pickled vegetables), spicy and greasy foods (e.g. Chinese hot pot), Chinese herbal medicine, and laxatives at least one month before stool collection.

### 2.3 Clinical data collection

A team of physicians from Guangdong Provincial Hospital of Chinese Medicine performed a face-to-face survey by using a health questionnaire that included demographics, medical history, medication usage, and behavioural risk factors. Subsequently, a team of nurses measured the heights and weights of the participants at the site and calculated their body mass indices (BMIs). Blood samples were collected after ≥10 h of fasting for analyses of the following clinical laboratory parameters: fasting glucose (GLU), total cholesterol (TC), triglycerides (TG), high-density lipoprotein cholesterol (HDL-C), low-density lipoprotein cholesterol (LDL-C), white blood cell (WBC), absolute neutrophil (NEUT), and hypersensitive C-reactive protein (hs-CRP). The participants underwent electrocardiography and carotid ultrasonography.

### 2.4 Carotid intima-media thickness measurement

In this study, the carotid intima-media thickness (IMT) was defined as the mean of the left and right IMTs of the common carotid artery (CCA) (Onut et al., 2012). A Doppler ultrasound (Philips, Netherlands) with a 3- to 12-MHz multifrequency linear probe was operated by a senior ultrasonographer in accordance with current clinical guidelines (The Ministry of Health China Stroke Prevention Project Committee, 2015; The Professional Committee of Vascular Ultrasound of Stroke Prevention and Treatment Expert et al., 2020). The IMT was scanned at the proximal far wall of the CCA (1–1.5 cm proximal to the carotid bifurcation and without plaque) (Stein et al., 2008). Subsequently, the vertical distance from the upper edge of the inner membrane to the upper edge of the outer membrane was measured.

### 2.5 Stroke risk stratification

The participants were primarily assessed using the Chinese National Stroke Risk Scorecard (CNSRS) (Chao et al., 2021) and divided into the following groups: healthy control group, participants without risk factors and a medical history of TIA/stroke; low-risk group, participants with <3 risk factors and without a medical history of hypertension, diabetes mellitus, or atrial fibrillation; moderate-risk group, participants with <3 risk factors and with hypertension, diabetes mellitus, or atrial fibrillation; and high-risk group, participants with ≥3 risk factors or a history of TIA/stroke.

Stroke risk was also stratified using the Framingham Stroke Risk Profile (FSRP) (D'Agostino et al., 1994), which is widely used to estimate the 10-year probability of incident stroke. FSRP included age, systolic blood pressure, cigarette smoking, cardiac disease (coronary heart disease, cardiac failure, intermittent claudication, atrial fibrillation, or left ventricular hypertrophy by electrocardiogram), diabetes mellitus, and anti-hypertensive medication use in the model, and its prediction was sex specific. The criteria in a previous study (Peng et al., 2020) were endorsed to define the risk level of FSRP. The participants were then divided into the following groups: low-risk group of FSRP, 10-Year Probability of stroke ≤ 5%; moderate-risk group, 5% < 10-Year Probability of stroke <15%; and high-risk group, 10-Year Probability of stroke ≥ 15%.

### 2.6 Stool sample collection, DNA extraction, and 16S ribosomal RNA gene sequencing

Stool samples were collected from the participants, stored at -80°C within 24 h, and then sent to BGI Genomics (Shenzhen, China) for sequencing. Bacterial DNA was extracted using the MagPure Stool DNA KF kit B (MD5115-01B, Magen, China) in accordance with the manufacturer’s instructions. The faecal microbiota composition was determined by sequencing the V4 region of the 16S rRNA gene on an Illumina HiSeq 2500 platform (BGI, Shenzhen, China) and generating 2×250 bp reads.

Raw reads were filtered to remove adaptors and low-quality and ambiguous bases, and paired-end reads were added to tags by using the Fast Length Adjustment of SHort reads program (FLASH, v1.2.11) (Magoč and Salzberg, 2011) to obtain the tags. The tags were clustered into operational taxonomical units (OTUs) with a cut-off value of 97% by using UPARSE software (v7.0.1090) (Edgar, 2013), and chimeric sequences were compared with those in the Gold database by using UCHIME (v4.2.40) (Edgar et al., 2011). OTU representative sequences were taxonomically classified using Ribosomal Database Project Classifier (v2.2) (Qiong et al., 2007), with a minimum confidence threshold of 0.6, and then trained on the Greengenes database v201305 by using QIIME v1.8.0 (Caporaso et al., 2010). All the tags were mapped back to the OTU sequences by using USEARCH_global (v7.0.1090) (Edgar, 2010) to obtain the OTU abundance statistical table for each sample.

### 2.7 Microbiome bioinformatics

The rarefaction curves for the observed species were plotted to determine whether the depth of sequencing adequately captured the full taxonomic diversity of all the samples (**Supplementary Figure 1**). Alpha and beta diversities were estimated at the OTU level by using MOTHUR (v1.31.2) (Schloss et al., 2009) and QIIME (v1.8.0) (Caporaso et al., 2010), respectively. The alpha diversity was determined by calculating the Chao1 (Chao, 1984), abundance-based coverage estimator (Ace) (Chao and Lee, 1992), Shannon (1948), and Simpson (1949) indices, whereas the beta diversity was evaluated by calculating the weighted and unweighted UniFrac distances (Lozupone et al., 2011). Linear discriminant analysis Effect Size (LEfSe) (Segata et al., 2011) analysis along with logarithmic linear discriminant analysis (LDA) scores > 2 and p values < 0.05 were considered in identifying the discriminative taxa. Kyoto Encyclopedia of Genes and Genomes (KEGG) (v77.1) functions were predicted using Phylogenetic Investigation of Communities by Reconstruction of Unobserved States (PICRUSt) (v2.3.0-b) (Wilkinson et al., 2018). The correlation between the outstanding gut microbiota and clinical characteristics was plotted using the R package pheatmap, and their correlation coefficients were calculated using Spearman’s correlation analysis. These results were visualised by preparing bar, box, and violin plots using R (version 4.2.2).

### 2.8 Statistical analysis

Statistical analyses were conducted using IBM SPSS Statistics for Windows version 26.0. Continuous variables are presented as mean (standard deviation or standard error of the mean) depending on the type of data distribution, and categorical variables are presented as numbers (percentages). For the continuous variables, either one-way ANOVA or Kruskal–Wallis H test was selected for multiple comparisons depending on the type of data distribution, and the Wilcoxon Rank Sum test was used for pairwise comparisons. For categorical data, the chi-square test was used for group comparisons. Spearman’s correlation analysis was used to explore the monotonic relationship between the continuous or ordinal data. Statistical significance was set at p < 0.05.

## 3 Results

### 3.1 Participant characteristics

**Figure 1:**
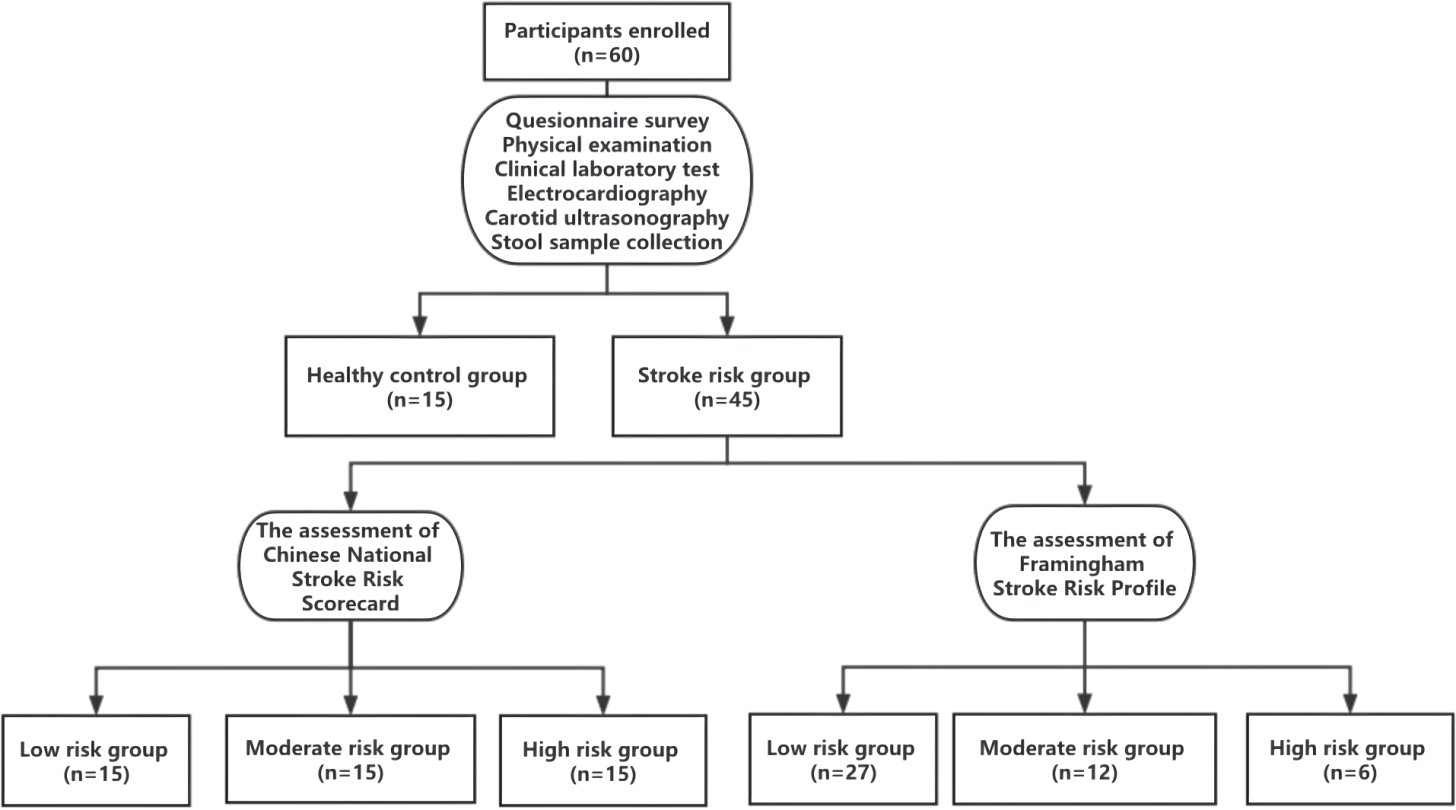
Study flowchart.

A total of 45 participants with stroke risk factors and 15 healthy controls were enrolled in the study between October 2021 and January 2022. The flowchart of the study is shown in **Figure 1**. The 60 participants had an average age of 52.58 years and a male/female ratio of 81.82%. None of the participants had a current or previous diagnosis of TIA/stroke. Only five participants had a diagnosis of cardiac disease or a family history of stroke, and physical inactivity was not identified by any of participants. No significant differences in age and cardiac disease were found between the participants from the healthy control group and CNSRS groups. However, no significant differences in overweight/obesity, dyslipidaemia, and family history of stroke were found between the participants from the FSRP groups. The demographic and clinical characteristics of the participants are detailed in **Table 1**.

**Table 1:** Basic characteristics of participants.

Characteristics	Chinese National Stroke Risk Scorecard					Framingham Stroke Risk Profile§			
	HC (n=15)	LR (n=15)	MR (n=15)	HR (n=15)	*p*	LR (n=27)	MR (n=12)	HR (n=6)	*p*
Age, year, mean (sd)	50.53 (7.220)	51.67 (5.627)	56.20 (7.093)	51.93 (7.245)	0.126	51.37 (4.732)	59.17 (8.111)	50 (6.197)	0.001*
Gender (Male, %)	3 (20%)	7 (46.7%)	3(20%)	14 (93.3%)	0.000*	10 (37.0%)	8 (66.7%)	6 (100%)	0.003*
Smoking, n (%)	0	5 (33.3%)	0	10 (66.7%)	0.000*	5 (18.5%)	5 (41.7%)	5 (83.3%)	0.008*
Overweight/obesity, n (%)	0	11 (73.3%)	9(60%)	11 (73.3%)	0.000*	19 (70.4%)	8 (66.7%)	4(66.7%)	0.966
Dyslipidemias, n (%)	0	5 (33.3%)	4 (26.7%)	12 (80%)	0.000*	12 (44.4%)	5 (41.7%)	4 (66.7%)	0.563
Hypertension, n (%)	0	0	12 (80%)	13 (86.7%)	0.000*	9 (33.3%)	11 (91.7%)	5 (83.3%)	0.001*
Diabetes, n (%)	0	0	3 (20%)	3 (20%)	0.029*	3 (11.1%)	0	3 (50%)	0.017*
Family history of stroke, n (%)	0	1 (6.7%)	0	4 (26.7%)	0.022*	3(11.1%)	2(16.7%)	0	0.418
Cardiac diseases, n (%)	0	1(6.7%)	2 (13.3%)	2 (13.3%)	0.319	0	2 (16.7%)	3 (50%)	0.002*
IMT, mm, mean (sd)	0.67(0.10)	0.70(0.09)	0.80(0.15)	0.90(0.17)	0.001*	0.77(0.14)	0.90(0.20)	0.82(0.76)	0.199
WBC, ×10^9^/L, mean (sd)	5.50(1.07)	5.42(0.77)	5.80(1.06)	7.53(1.69)	0.000*	5.69(1.00)	6.99(1.94)	7.33(1.49)	0.005*
NEUT, ×10^9^/L, mean (sd)	3.45(1.01)	2.97(0.48)	3.37(0.77)	4.56(1.03)	0.001*	3.13(0.62)	4.24(1.11)	4.62(1.04)	0.001*
TG, mmol/L, mean (sd)	1.11(0.25)	2.05(1.79)	1.20(0.35)	2.16(1.42)	0.025*	1.76(1.39)	1.39(0.72)	2.66(1.78)	0.209
TC, mmol/L, mean (sd)	5.36(0.60)	4.89(0.69)	5.05(0.63)	4.88(0.85)	0.957	5.29(0.80)	4.57(0.36)	4.60(0.41)	0.063
LDL-C, mmol/L, mean (sd)	3.42(0.39)	3.10(0.64)	3.22(0.48)	3.18(0.59)	0.949	3.34(0.69)	3.00(0.29)	2.99(0.30)	0.482
HDL-C, mmol/L, mean (sd)	1.55(0.40)	1.24(0.23)	1.47(0.20)	1.09(0.24)	0.000*	1.32(0.23)	1.25(0.35)	1.09(0.18)	0.080
hs-CRP, mg/L, mean (sd) ^#^	0.73 (0.57)	0.73 (0.31)	1.22 (1.28)	2.38 (2.07)	0.530	0.95 (0.97)	2.14 (1.87)	0.65(0.16)	0.219
GLU, mmol/L, mean (sd)	4.90(0.43)	5.18(0.72)	6.48(2.60)	6.39(2.82)	0.023*	5.83(2.10)	5.53(0.86)	7.87(4.04)	0.588

HC, healthy control group; LR, low-risk group; MR, moderate-risk group; HR, high-risk group; IMT, intima-media thickness; WBC, white blood cell; NEUT, total neutrophils; TG, triglycerides; TC, total cholesterol; LDL-C, low-density lipoprotein cholesterol; HDL-C, high-density lipoprotein cholesterol; hs-CRP, hypersensitive C-reactive protein; GLU, fasting glucose. § Healthy controls were excluded from the samples assessed using the Framingham Stroke Risk Profile. ^#^ The hs-CRP was only detected in 17 samples. * p value < 0.05. We did not present the status of physical inactivity as it was not identified in any of the participants.

### 3.2 Gut microbiome between the CNSRS groups and healthy control group

**Figure 2:**
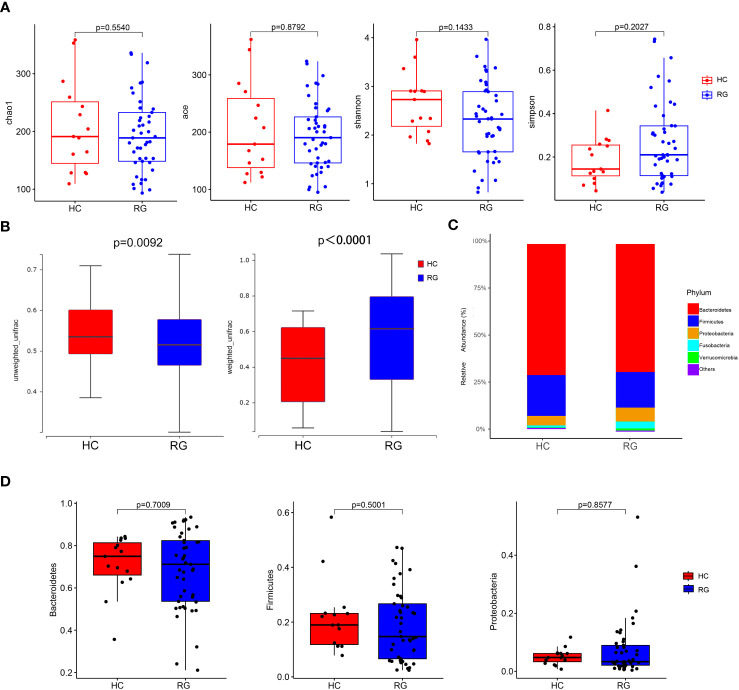
Comparison of microbial diversity and composition between the CNSRS risk groups and the healthy control group. **(A)** No significant difference was found in alpha diversity based on the Chao1, Ace, Shannon, and Simpson indices (p > 0.05). **(B)** A significant difference in beta diversity was found in terms of weighted (p < 0.0001) and unweighted UniFrac distances (p = 0.0092). **(C)** Relative abundance distribution of five dominant phyla of bacteria. **(D)** Relative abundances of *Bacteroidetes*, *Firmicutes*, and *Proteobacteria* did not differ significantly between the groups by the Wilcoxon test. The relative abundances of *Fusobacteria* and *Verrucomicrobia* were not detected in 95% samples and not significantly different between RG and HC (p = 0.1841, p = 0.4063, respectively). CNSRS, Chinese National Stroke Risk Scorecard; HC, healthy control group; RG, risk group.

The OTU dataset for the CNSRS groups and healthy control group comprised 769 OTUs that classified 140 genera, 54 families, and 13 phyla. No significant differences in alpha diversity indices (Chao1, Ace, Shannon, and Simpson) were identified between the CNSRS groups and healthy control group (**Figure 2A**). By contrast, significant differences in beta diversity were observed between the groups in terms of weighted (p < 0.0001) and unweighted (p = 0.0092) UniFrac distances (**Figure 2B**). The five dominant phyla in the microbiota of all participants were *Bacteroidetes* (69.0548% ± 2.2443%), *Firmicutes* (19.6341% ± 1.6876%), *Proteobacteria* (6.8188% ± 1.1084%), *Fusobacteria* (2.9961% ± 1.2420%), and *Verrucomicrobia* (0.9263% ± 0.7835%) (**Figure 2C**), and their relative abundances were not significantly different between the CNSRS groups and healthy control group (p > 0.05, **Figure 2D**).

**Figure 3:**
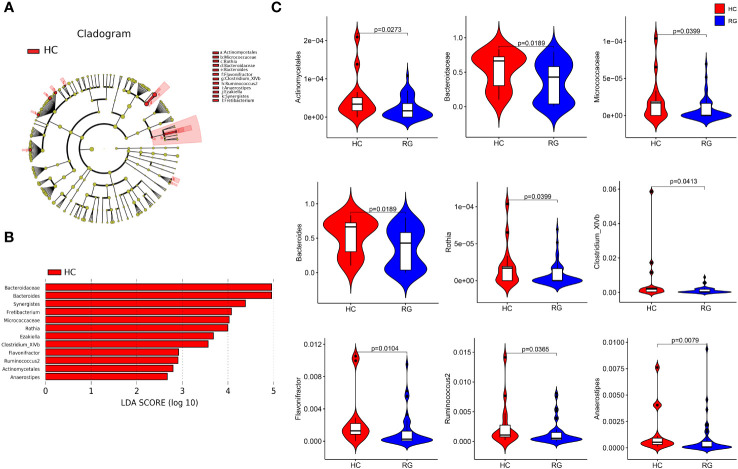
Discriminative taxa between the CNSRS risk groups and the healthy control group. **(A)** Cladogram showing the taxa with different abundance values based on LEfSe analysis. **(B)** Discriminative taxa between RG and HC were determined using LEfSe analysis along with LDA scores (log10) > 2 and p < 0.05. The red bar chart represents the bacteria that were more abundant in HC than RG. **(C)** Relative abundances of *Bacteroidaceae*, *Bacteroides*, *Micrococcaceae*, *Rothia*, *Clostridium*_*XlVb*, *Flavonifractor*, Ruminococcus 2, *Actinomycetales*, and *Anaerostipes* were significantly different between RG and HC (p < 0.05) as determined using the Wilcoxon test. The relative abundances of *Synergistes*, *Fretibacterium* and *Ezakiella* were not detected in more than 95% samples but were significantly different between RG and HC (p = 0.015, p = 0.003, p = 0.015, respectively). CNSRS, Chinese National Stroke Risk Scorecard; LEfSe, linear discriminant analysis effect size; LDA, linear discriminant analysis; HC, healthy control group; RG, risk group.

The LEfSe analysis with an LDA score of more than 2 points identified one bacterial order (*Actinomycetales*), two bacterial families (*Bacteroidaceae* and *Micrococcaceae*), and nine bacterial genera (*Bacteroides*, *Rothia*, *Synergistes*, *Fretibacterium*, *Ezakiella*, *Clostridium_XlVb*, *Flavonifractor*, *Ruminococcus 2*, and *Anaerostipes*), which were all enriched in the participants from the healthy control group, and their relative abundances were significantly different between the participants from the healthy control and CNSRS groups (**Figure 3**). The flora with the highest abundance included *Bacteroidaceae* at the family level and *Bacteroides* at the genus level.

### 3.3 Gut microbiota in different CNSRS groups

**Figure 4:**
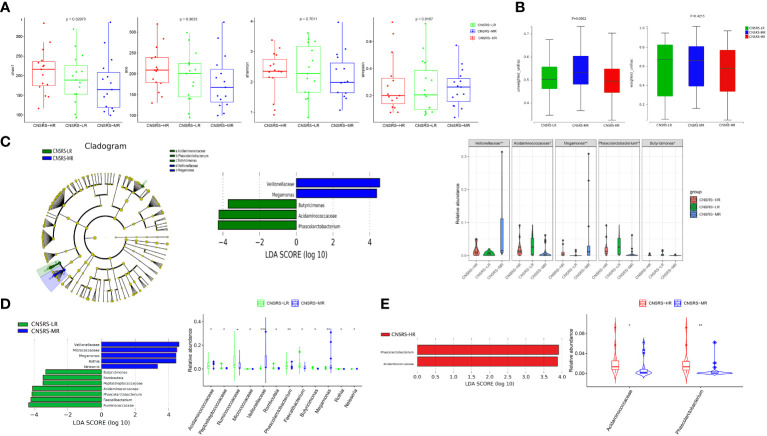
Microbial diversity and composition in different CNSRS groups. **(A)** No significant difference in alpha diversity was found in terms of the Chao1, Ace, Shannon, and Simpson indices (p > 0.05). **(B)** Significant difference in beta diversity was found among the participants from the three CNSRS groups in terms of unweighted UniFrac distances (p = 0.0002), whereas no apparent difference was found in terms of weighted UniFrac distances (p = 0.4213). **(C)** Cladogram showing the taxa with different abundance values based on LEfSe analysis. Discriminative taxa between groups were determined using LEfSe analysis along with LDA scores (log10) > 2 and p < 0.05. Green bar chart represents the bacteria enriched in LR, and the blue bar chart represents the bacteria enriched in MR. Kruskal–Wallis test results suggested that the relative abundances of *Veillonellaceae*, *Acidaminococcaceae*, *Megamonas*, *Phascolarctobacterium*, and *Butyricimonas* were significantly different among the participants from the three CNSRS groups. **(D)** LEfSe analysis along with LDA scores was applied in pairwise comparisons. The green bar chart represents the bacteria enriched in LR, and the blue bar chart represents the bacteria enriched in MR in the pairwise comparisons between LR and Wilcoxon Rank Sum test suggested that the relative abundances of 12 taxa were significantly different between the participants from LR and MR. **(E)** Red bar chart represents the bacteria enriched in HR in the pairwise comparisons between MR and HR. Wilcoxon Rank Sum test suggested that the relative abundances of two taxa were significantly different between the participants from LR and MR. CNSRS, Chinese National Stroke Risk Scorecard; LEfSe, linear discriminant analysis effect size; LDA, linear discriminant analysis; HR, high-risk group; LR, low-risk group; MR, moderate-risk group. p values: *p < 0.05, ^**^p < 0.01, ^***^p < 0.001.

No significant difference in alpha diversity was detected among the three CNSRS groups (**Figure 4A**). Meanwhile, a significant difference in beta diversity was found among the three groups in terms of unweighted UniFrac distance (p = 0.0002, **Figure 4B**) but none in terms of weighted UniFrac distance (p = 0.4213, **Figure 4B**).

LEfSe analysis along with LDA scores identified two bacterial families (*Veillonellaceae* and *Acidaminococcaceae*) and three bacterial genera (*Megamonas*, *Phascolarctobacterium*, and *Butyricimonas*), which were statistically different among the three groups (**Figure 4C**). *Acidaminococcaceae*, *Phascolarctobacterium* and *Butyricimonas* were enriched in the participants from the low-risk group, whereas *Veillonellaceae* and *Megamonas* were enriched in the participants from the moderate-risk group.

Furthermore, pairwise comparisons between the participants from the low- and moderate-risk groups showed that *Veillonellaceae*, *Megamonas*, *Micrococcaceae*, *Rothia*, and *Neisseria* were enriched in the participants from the moderate-risk group, whereas *Peptostreptococcaceae*, *Romboutsia*, *Acidaminococcaceae*, *Phascolarctobacterium*, *Ruminococcaceae*, *Faecalibacterium*, and *Butyricimonas* were enriched in the participants from the low-risk group (**Figure 4D**). Pairwise comparisons between the participants from the moderate- and high-risk groups revealed that *Acidaminococcaceae* and *Phascolarctobacterium* were enriched in the participants from the high-risk group (**Figure 4E**).

### 3.4 Predictive functional profiling of microbial communities and associations with CNSRS

**Figure 5:**
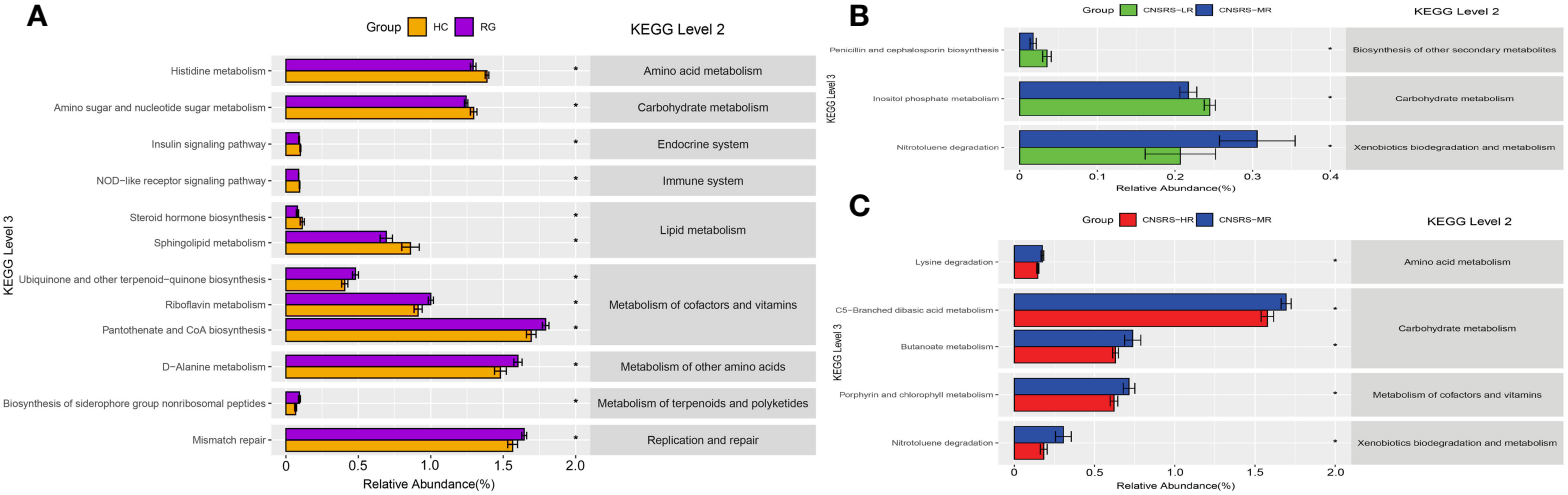
KEGG pathways predicted by PICRUSt and associations with CNSRS. **(A)** KEGG pathways with differences in abundance between RG and HC. **(B)** KEGG pathways with differences in abundance between LR and MR. **(C)** KEGG pathways with differences in abundance between MR and HR. CNSRS, Chinese National Stroke Risk Scorecard; KEGG, Kyoto Encyclopedia of Genes and Genomes; PICRUSt, Phylogenetic Investigation of Communities by Reconstruction of Unobserved States; HC, healthy control group; HR, high-risk group; LR, low-risk group; MR, moderate-risk group; RG, risk group. Differential KEGG pathways with relative abundance were visualized as bar plot with standard error. *Wilcoxon Rank Sum test with p values less than 0.05.

Functional profiles of microbial communities predicted by PICRUSt identified 14 KEGG pathways in which the microbial abundance of the participants from the CNSRS groups was significantly lower than that of the healthy controls (p < 0.05). After removing pathways with low occurrence and abundance, 12 KEGG pathways remained. These pathways were related to the metabolism of cofactors, vitamins, lipids, terpenoids, polyketides, amino acids, and carbohydrates and to the endocrine system, immune system, replication, and repair. **Figure 5A** shows the matching relationships of KEGG pathways between levels 2 and 3 and the group comparison of microbial abundance for each pathway.

Furthermore, pairwise comparisons between the participants from the low- and moderate-risk groups revealed three pathways in which the microbial abundance was significantly different (p < 0.05). These pathways were related to penicillin and cephalosporin biosyntheses (1 pathway), carbohydrate metabolism (1 pathway), and xenobiotic biodegradation and metabolism (1 pathway) (**Figure 5B**). Meanwhile, pairwise comparisons between the participants from the moderate- and high-risk groups revealed five outstanding pathways (p < 0.05). These pathways were involved in carbohydrate metabolism (2 pathways), amino acid metabolism (1 pathway), cofactor and vitamin metabolism (1 pathway), and xenobiotic biodegradation and metabolism (1 pathway) (**Figure 5C**).

### 3.5 Gut microbial diversity and composition in different FSRP groups

**Figure 6:**
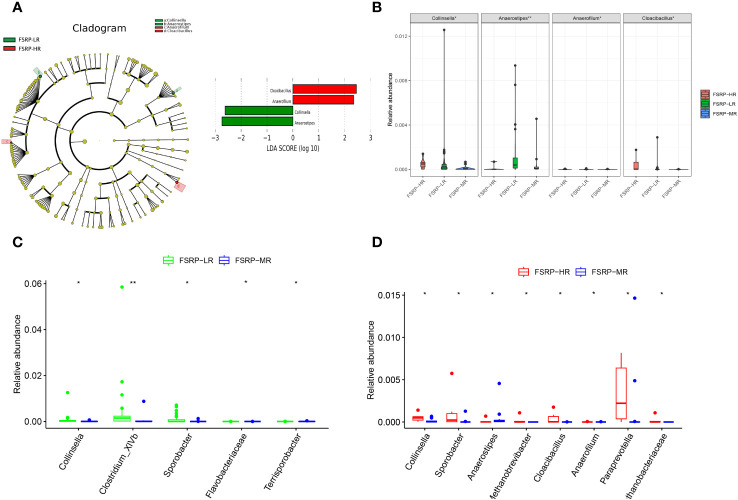
Discriminative taxa among different FSRP groups. **(A)** Cladogram reporting the taxa with different abundance values based on LEfSe analysis. Discriminative taxa between groups were determined using LEfSe analysis along with LDA scores (log10) > 2 and p < 0.05. The green bar chart represents the bacteria enriched in LR, and the red bar chart represents the bacteria enriched in HR. **(B)** Kruskal–Wallis test results suggested that the relative abundances of *Collinsella*, *Anaerostipes*, *Anaerofilum* and *Cloacibacillus* were significantly different among the three FSRP groups. **(C)** Wilcoxon Rank Sum test suggested that the relative abundances of five taxa were significantly different between LR and MR. **(D)** Wilcoxon Rank Sum test suggested that the relative abundances of eight taxa were significantly different between MR and HR. FSRP, Framingham Stroke Risk Profile; LEfSe, linear discriminant analysis effect size; LDA, linear discriminant analysis; HR, high-risk group; LR, low-risk group; MR, moderate-risk group. p values: *p < 0.05, ^**^p < 0.01.

No significant differences in either alpha or beta diversity were identified among the participants from the different FSRP groups. LEfSe analysis along with LDA scores suggested that *Collinsella* and *Anaerostipes* were enriched in the participants from the low-risk group, whereas *Anaerofilum* and *Cloacibacillus* were enriched in the participants from the high-risk group (**Figure 6A**). The relative abundances of these genera were significantly different among the three FSRP groups (**Figure 6B**).

Furthermore, pairwise comparisons between the participants from the low- and moderate-risk groups suggested that *Collinsella*, *Clostridium_XlVb*, and *Sporobacter* were enriched in the participants from the low-risk group, whereas *Flavobacteriaceae* and *Terrisporobacter* were enriched in the participants from the moderate-risk group (**Figure 6C**). Pairwise comparisons between the participants from the moderate- and high-risk groups revealed that *Methanobacteriaceae*, *Collinsella*, *Sporobacter*, *Cloacibacillus*, *Paraprevotella*, *Anaerofilum*, and *Methanobrevibacter* were enriched in the participants from the high-risk group, whereas *Anaerostipes* was enriched in the participants from the moderate-risk group (**Figure 6D**). Although the between-group difference was statistically significant, the discriminative microbiota determined by pairwise comparisons was marginally abundant.

### 3.6 Predictive functional profiling of microbial communities and associations with FSRP

**Figure 7:**
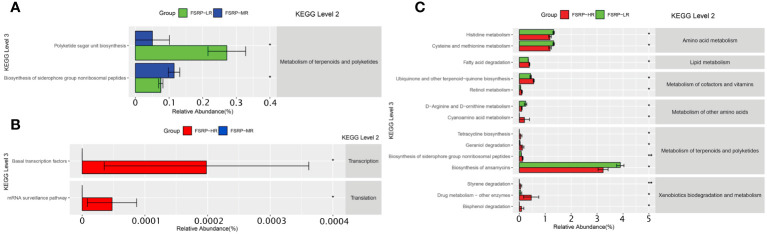
KEGG pathways predicted by PICRUSt and associations with FSRP groups. **(A)** KEGG pathways with differences in abundance between LR and MR. **(B)** KEGG pathways with differences in abundance between MR and HR. **(C)** KEGG pathways with differences in abundance between LR and HR. FSRP, Framingham Stroke Risk Profile; KEGG, Kyoto Encyclopedia of Genes and Genomes; PICRUSt, Phylogenetic Investigation of Communities by Reconstruction of Unobserved States; HR, high-risk group; LR, low-risk group; MR, moderate-risk group. Differential KEGG pathways with relative abundance were visualized as a bar plot with standard error. *Wilcoxon Rank Sum test with p values less than 0.05, ** Wilcoxon Rank Sum test with p values less than 0.01.

The KEGG pathways of microbial communities associated with the risk levels of FSRP were further investigated using PICRUSt. Pairwise comparisons between the participants from the low- and moderate-risk groups revealed two outstanding pathways related to the metabolism of terpenoids and polyketides (**Figure 7A**). Pairwise comparisons between the participants from the moderate- and high-risk groups showed two outstanding pathways associated with transcription and translation (**Figure 7B**). Pairwise comparisons between the participants from the low- and high-risk groups demonstrated 14 KEGG (level 3) pathways of significant difference. These pathways were involved in the metabolism of amino acids, lipids, cofactors, vitamins, terpenoids, and polyketides and in the biodegradation and metabolism of xenobiotics (**Figure 7C**).

### 3.7 Correlation between the gut microbiota and clinical characteristics

**Figure 8:**
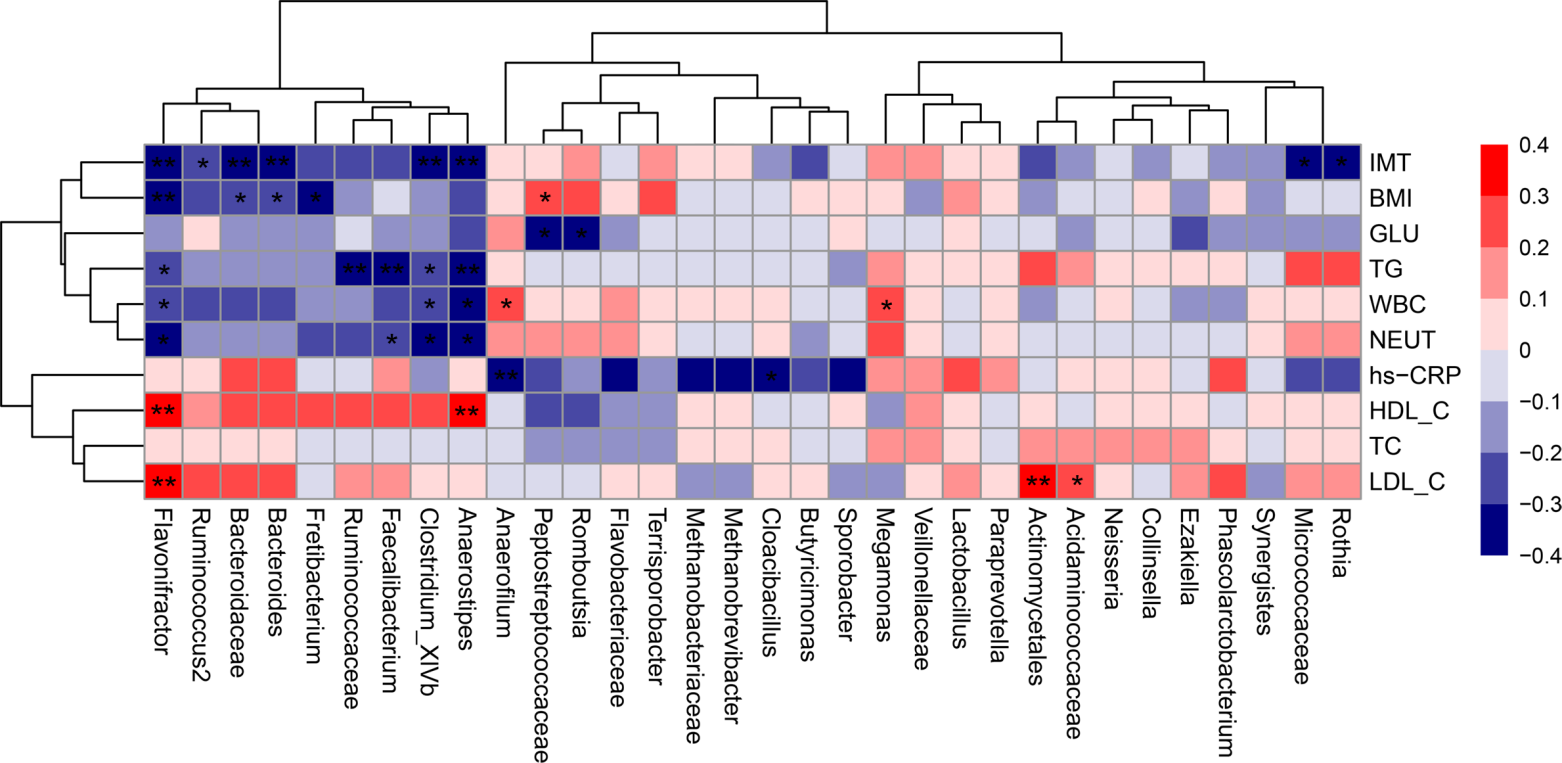
Correlation matrix for discriminative microbiota and clinical characteristics. Red cell indicates positive correlation, and blue indicates negative correlation in the Spearman correlation analysis. ^*^p < 0.05, ^**^p < 0.01. BMI, body mass index; GLU, fasting glucose; HDL-C, high-density lipoprotein cholesterol; hs-CRP, hypersensitive C-reactive protein; IMT, intima-media thickness; LDL-C, low-density lipoprotein cholesterol; NEUT, total neutrophils; TC, total cholesterol; TG, triglycerides; WBC, white blood cell.

Spearman rank-order analysis of the correlation between the discriminative gut microbiota and clinical characteristics (**Figure 8**) showed that *Anaerostipes*, *Flavonifractor* and *Clostridium_XlVb* were negatively correlated with IMT, TG, WBC, and NEUT, whereas *Anaerostipes* and *Flavonifractor* were positively correlated with HDL-C. *Faecalibacterium* was negatively correlated with TG and NEUT, and *Ruminococcus2*, which is under the same phylum (*Firmicutes*) as *Faecalibacterium*, showed a negative monotonic relationship with IMT. In addition, *Bacteroidaceae* and *Bacteroides* were negatively correlated with IMT and BMI, and Cloacibacillus were negatively correlated with hs-CRP. Correlation analysis also identified some gut microbiota specific to individual risk factors; for example, *Peptostreptococcaceae* and *Romboutsia* specifically benefited to the alteration of glucose, *Fretibacterium* negatively correlated with BMI, and *Ruminococcaceae* prevented the elevation of TG. In addition to beneficial bacteria, some pathogenic bacteria were identified; for example, *Actinomycetales* and *Acidaminococcaceae* contributed to the enhancement of LDL-C, whereas *Megamonas* and *Anaerofilum* contributed to the increase in WBC; Anaerofilum was also negatively correlated with hs-CRP.

## 4 Discussion

In the present study, many vascular beneficial bacteria were enriched in the participants from the healthy control and low-risk stroke groups, whereas a few vascular harmful bacteria were more abundant in the participants from the high-risk group than in those from the other groups. However, Zeng et al. (2019) assessed southern participants with a mean age of 71 years based on CNSRS and found that opportunistic pathogens and lactate-producing bacteria are enriched in those participants with a high risk of stroke (Zeng et al., 2019). This result may be partially explained by the age difference between the participants of the two studies, as microbial diversity and composition are altered in elderly people (Vaiserman et al., 2017). Specifically, Rondanelli et al. (2015) reported that the population of *Enterobacteriaceae* increases in people aged more than 65 years (Rondanelli et al., 2015). This family was also detected in the study by Zeng et al. (2019) but not in the present study. Meanwhile, Rondanelli et al. (2015) found that the populations of *Bacteroides* and *Clostridium* are greatly reduced in elderly people (Rondanelli et al., 2015). These genera were enriched in the healthy controls of our study but not identified in the participants from the study by Zeng et al. (2019). These differences may be also ascribed to the sex difference between the studies because emerging evidence suggests that sex–microbiota–brain interactions are involved in the development of neurologic and psychiatric disorders (Shobeiri et al., 2022). Specifically, laboratory experiments showed that *Lactobacillus* is generally more enriched in females than in males (Shobeiri et al., 2022). Similarly, Zeng et al. (2019) found that *Lactobacillus* is enriched in a high-risk group composed of 58% females. However, *Lactobacillus* was not identified in the present study, where females only accounted for approximately 7% of the high-risk group. Our study also found that the participants in the low-risk group had an abundance of butyrate-producing bacteria, such as *Faecalibacterium*, *Ruminococcaceae*, *Butyricimonas*, and *Peptostreptococcaceae*. This finding is consistent with the results reported by Zeng et al. (2019). Both studies suggested that the accumulation of inflammation is correlated with the elevated stroke risk, considering that butyrate-producing bacteria function as anti-inflammatory and anti-atherosclerotic agents (Zhao et al., 2021).

The gut microbiota is a promising biomarker for predicting atherosclerotic cardiovascular diseases (Nayor et al., 2021). Our study revealed that *Anaerostipes*, *Clostridium_XlVb*, and *Flavonifractor*, all genera belonging to the *Firmicutes* phylum, were enriched in the participants from the low-risk group as assessed by CNSRS or FSRP, and their relative abundances gradually decreased as the stroke risk increased. In addition, the gut microbiota has a negative monotonic relationship with IMT, a widely used surrogate marker for atherosclerosis (Nezu et al., 2016). This finding deserves attention as it provides specific implications for developing novel microbial biomarkers for predicting stroke risk in future cohort studies. Furthermore, we found that *Anaerostipes*, *Clostridium_XlVb*, and *Flavonifractor* were negatively correlated with TG. Although elevated TG levels might not directly lead to atherosclerosis, triglyceride-rich lipoproteins (TGRL) and remnant lipoprotein particles (RLP) are causal risk factors for atherosclerotic cardiovascular events because of several reasons: 1) TGRL remnants can penetrate the endothelium and interact with macrophages, leading to foam cell formation and inflammation in arterial walls; 2) TGRL can be absorbed by circulating monocytes and become lipid-laden foamy monocytes, which infiltrate the arterial wall; and 3) TGRL and RLP can induce HDL remodelling and dysfunction, all of which promote vascular inflammation and atherosclerosis (Peng and Wu, 2022). The negative association between the gut microbiota and TG may contribute to the development of novel therapies, such as supplemental probiotics targeting hypertriglyceridaemia. In addition, *Anaerostipes*, *Clostridium_XlVb*, and *Flavonifractor* were positively correlated with HDL-C, whereas *Flavonifractor* was positively correlated with LDL-C. The benefits of these microbiota in atherosclerosis are supported by the traditional knowledge that HDL-C, the plasma lipoprotein responsible for reverse cholesterol transport, protects against atherosclerosis (Fernandes das Neves et al., 2021). However, the positive correlation of *Flavonifractor* with LDL-C was unexpected, considering that elevated LDL-C is a well-established risk factor for cardiovascular disease (Silverman et al., 2016). This finding may be explained by the fact that an increase in circulating oxidised LDL-C (oxLDL-C) is the primary contributor to the induction of foam cells in the arterial intima and that plasma oxLDL-C level is a better predictor of cardiovascular disease than LDL-C level (Zhang et al., 2019; Xu et al., 2022). We also found that *Anaerostipes*, *Clostridium_XlVb*, and *Flavonifractor* were negatively correlated with WBC and NEUT counts, which are universally available markers of chronic low-grade inflammation that are associated with carotid IMT and plaques presented in early and advanced atherosclerosis (Ortega et al., 2012; Willeit et al., 2016). Taken together, these findings suggest that *Anaerostipes*, *Clostridium_XlVb*, and *Flavonifractor* protect against atherosclerosis and reduce the risk of incident stroke by reducing lipid accumulation and attenuating the inflammatory responses of the arterial walls, which generally was consistent with the functional prediction results.

Additionally, the genus *Bacteroides* and its family *Bacteroidaceae* with a higher abundance in the healthy control group than in the stroke risk groups, were found negatively correlated with BMI, which were in line with the results of a previous study (Liu et al., 2017). Their negatively correlation with IMT might be attributed to their attenuating effect on atherosclerotic lesion by suppressing pro-inflammatory immune responses (Yoshida et al., 2018). Apart from the beneficial vascular microbiota, we also identified *Megamonas* and *Anaerofilum*, which were enriched in the participants from the high-risk group and positively correlated with WBC count. The identification of vascular harmful microbiota is supported by the previous findings that *Megamonas* enriched in a group of coronary artery diseases is positively correlated with cholesterol (Liu et al., 2019) and that the population of *Anaerofilum* increases in people consuming large amounts of ultra-processed foods, which promote inflammation possibly via diet–microbiome–host interactions (Zinöcker and Lindseth, 2018; Cuevas-Sierra et al., 2021).

The present study used CNSRS and FSRP in the assessment of stroke risk and characterised the gut microbiota by risk levels for the first time. The CNSRS, which was developed by Chinese government, has been used nationwide owing to its feasibility in the rapid determination of stroke risk and its inclusion of recently identified risk factors, such as obesity and dyslipidaemia (Chao et al., 2021). Using CNSRS, we found outstanding vascular beneficial bacteria, such as *Phascolarctobacterium* and *Faecalibacterium*, which ameliorate hyperlipidaemia and inflammation (Sokol et al., 2008; Tong et al., 2018; Oliver et al., 2022), and *Bacteroides*, which attenuates carotid atherosclerosis by altering BMI (Liu et al., 2017). These pieces of evidence can be directly generalised to the Chinese population, considering that obesity and metabolic conditions are leading risk factors for stroke (Ma et al., 2021; Pan et al., 2021). FSRP has long been used globally because it considers age, sex, systolic blood pressure, and anti-hypertensive medication to calculate stroke risk, and the 10-year stroke risk model is validated by a large-sample, long-follow-up cohort study (Peng et al., 2020). Using FSRP, we identified outstanding microbiota, such as *Anaerostipes* and *Clostridium_XlVb*, which reportedly lower blood pressure (Bier et al., 2018; Verhaar et al., 2020). In addition, *Clostridium_XlVb* produces indole-3-propionic acid, which reverses cholesterol transport for atherosclerosis (Xue et al., 2022). The mutual complementation of the two stroke risk instruments broadens our understanding of the microbial diversity and composition in terms of stroke risk levels and offers candidates for future development of probiotics in cardiovascular medicine.

This study had several limitations. Firstly, the evidence certainty on the association between the gut microbiota and the risk of stroke may be limited by the small-scale, cross-sectional, single-centre design of the present study. Future cohort studies with large sample sizes, multiple sites, and long-term follow-up are needed to validate these findings. Secondly, we did not conduct metagenomic sequencing and detect the microbial metabolites. The functional prediction and explanations of the mechanisms of actions of the enriched microbiota should be further elucidated. Thirdly, the carotid plaque scores were not calculated in the present study. Such scores should be calculated because they represent atherosclerotic progress and predict stroke risk more accurately than carotid IMT (Nezu and Hosomi, 2020; Raggi and Stein, 2020).

## 5 Conclusion

The preliminary evidence suggests that gut microbiota is associated with stroke risk stratification, and it ameliorates atherosclerotic progress possibly by regulating lipid metabolism and inflammation. This study provides novel insights into the microbial characteristics related to stroke risk levels in middle-aged individuals, which is critical to the early screening of stroke risk and primary prevention.

## Data availability statement

According to national legislation/guidelines, specifically the Administrative Regulations of the People’s Republic of China on Human Genetic Resources (http://www.gov.cn/zhengce/content/2019-06/10/content_5398829.htm, http://english.www.gov.cn/policies/latest_releases/2019/06/10/content_281476708945462.htm), no additional raw data is available at this time. Data of this project can be accessed after an approval application to the China National Genebank (CNGB, https://db.cngb.org/cnsa/). Please refer to https://db.cngb.org/, or email: CNGBdb@cngb.org for detailed application guidance. The accession code CNP0005062 should be included in the application.

## Author contributions

XN conceived and designed the study, supervised its implementation, validated the data, and wrote the manuscript. HH led the study implementation, data analysis, and visualisation. ZK, RM, and MM participated in the survey, sample collection, and data entry. YC participated in participant recruitment and diagnosis ascertainment. XN and HH contributed to the interpretation of results. All the authors have read the manuscript and approved its submission.

## Supplementary material

Supplementary Figure 1 The rarefaction curves for observed species. HC, healthy control group; RG, risk group.
