# Supplementary figures and images for: The preliminary evidence on the association of the gut microbiota with stroke risk stratification in South Chinese population
Source: Front Cell Infect Microbiol. 2023 Dec 21;13:1227450. doi: 10.3389/fcimb.2023.1227450 (PMC10785002; doi:10.3389/fcimb.2023.1227450)

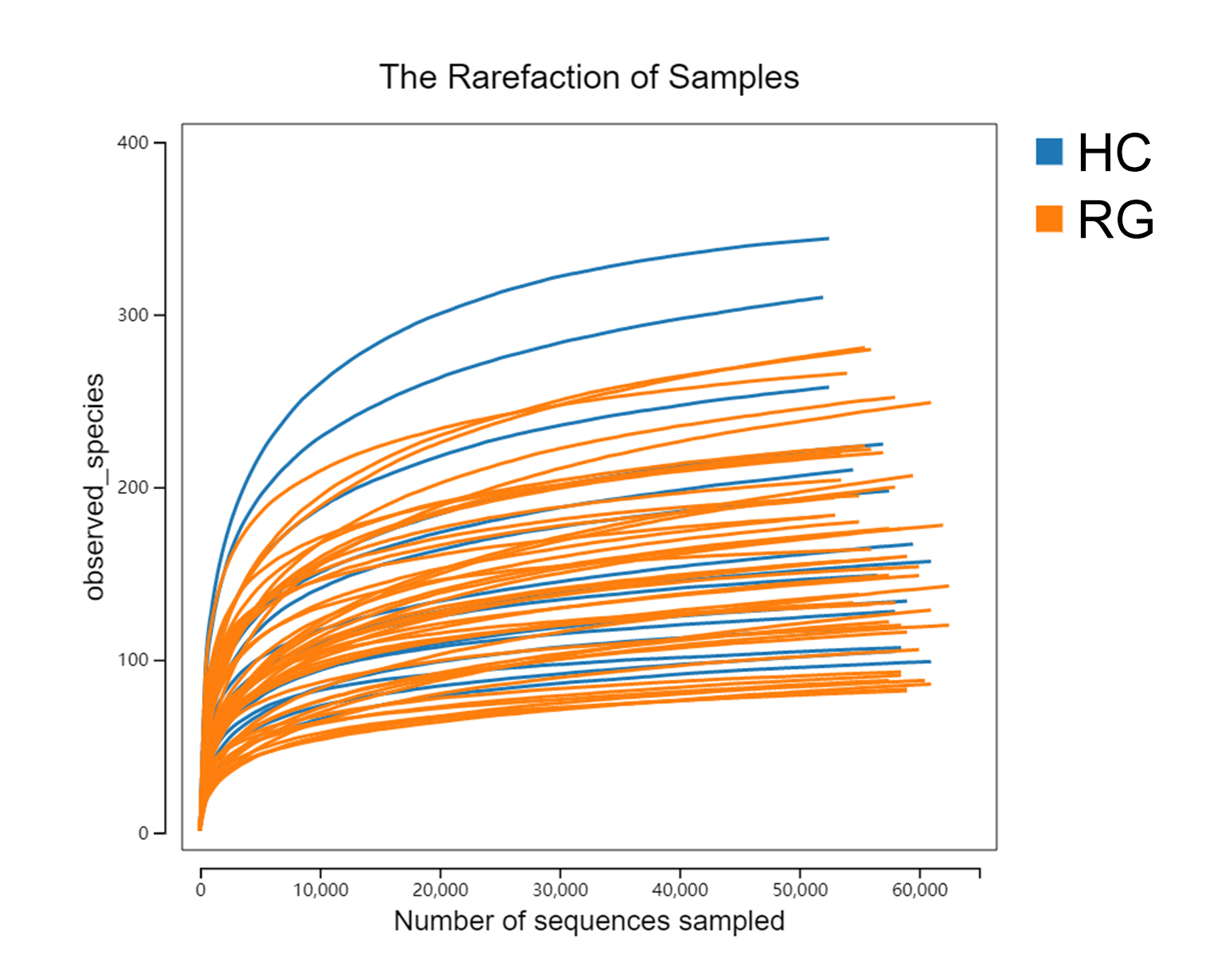

Supplement: Supplementary Figure 1 — The rarefaction curves for observed species. HC, healthy control group; RG, risk group. [file Image_1.tif]
