# Supplementary material for: The preliminary evidence on the association of the gut microbiota with stroke risk stratification in South Chinese population
Source: Front Cell Infect Microbiol. 2023 Dec 21;13:1227450. doi: 10.3389/fcimb.2023.1227450 (PMC10785002; doi:10.3389/fcimb.2023.1227450)
Supplement: Supplementary file 2 [file Table_1.docx]

**Table S1. Diagnostic criteria of stroke risk factors**

| **Risk factor/disease** | **Criteria** |
| --- | --- |
| **Hypertension** | 1. A medical history of hypertension with/without treatment, or 2. Systolic blood pressure ≥140 mmHg and/or diastolic blood pressure ≥90 mmHg, which occurs more than three times in different days. |
| **Diabetes mellitus** | 1. A medical history of diabetes mellitus with/without treatment, or 2. Fasting plasma glucose ≥7.0 mmol/L in the current study |
| **Atrial fibrillation** | 1. A medical history of atrial fibrillation, with/without treatment, or 2. Atrial fibrillation identified by the ECG in the current study |
| **Dyslipidemia** | 1. A medical history of dyslipidemia with/without treatment, or 2. With one or more than one item as follows in the current study: total cholesterol≥6.22 mmol/L (240 mg/dl), triglycerides ≥2.3 mmol/L (200 mg/dl), HDL-C<1.04 mmol/L (40 mg/dl), LDL-C ≥4.1 mmol/L (160 mg/dl) |
| **Overweight/obesity** | 1. Current body mass index (BMI) ≥24 kg/m^2^ defined as overweight, 2. Current body mass index (BMI) ≥28 kg/m2 defined as obesity,   **Notes:** BMI was calculated from weight in kilograms divided by height in meters squared. |
| **Smoking** | 1. Current smoking, or 2. Current smoking cessation while smoking ≥ 6 months in the past |
| **Physical inactivity** | 1. Physical exercise <3 times/week and lasting 30 minutes each time, 2. Industrial and agricultural labor considered as physical activity. |
| **Family history of stroke** | 1. One or more than one of family member with a history of stroke, 2. Reported by the participants in the survey, and 3. The family member limited to parents, brothers and sisters |
| **Transient ischemic attack (TIA) or stroke** | 1. A medical history of TIA or stroke, and 2. With supporting imaging records |
